# A Comparative Study of Structural Deformation Test Based on Edge Detection and Digital Image Correlation

**DOI:** 10.3390/s23083834

**Published:** 2023-04-08

**Authors:** Ruixiang Tang, Wenbing Chen, Yousong Wu, Hongbin Xiong, Banfu Yan

**Affiliations:** 1 School of Civil Engineering, Hunan University, Changsha 410082, China; 2 College of Civil and Architectural Engineering, Guangxi University, Nanning 530004, China

**Keywords:** deformation test, digital image processing, digital image correlation, Canny, Zernike moments

## Abstract

Digital image-correlation (DIC) algorithms rely heavily on the accuracy of the initial values provided by whole-pixel search algorithms for structural displacement monitoring. When the measured displacement is too large or exceeds the search domain, the calculation time and memory consumption of the DIC algorithm will increase greatly, and even fail to obtain the correct result. The paper introduced two edge-detection algorithms, Canny and Zernike moments in digital image-processing (DIP) technology, to perform geometric fitting and sub-pixel positioning on the specific pattern target pasted on the measurement position, and to obtain the structural displacement according to the change of the target position before and after deformation. This paper compared the difference between edge detection and DIC in accuracy and calculation speed through numerical simulation, laboratory, and field tests. The study demonstrated that the structural displacement test based on edge detection is slightly inferior to the DIC algorithm in terms of accuracy and stability. As the search domain of the DIC algorithm becomes larger, its calculation speed decreases sharply, and is obviously slower than the Canny and Zernike moment algorithms.

## 1. Introduction

Structural health monitoring has always been an important research direction in the field of civil engineering [1,2]. Damage detection and performance evaluation of the structures are crucial to reduce maintenance costs [3,4]. Displacement monitoring is an important parameter for structural health monitoring, and the computer vision-based structural displacement measurement method has the advantages of non-contact, high accuracy, and long-term monitoring. Commonly used computer vision-based displacement measurement methods include DIC (digital image correlation) and DIP (digital image processing) [5].

DIP-based non-contact measurement originates from computer vision technology and is generally applied in scenarios where factory-accurate dimensional measurements are required, such as in mechanical manufacturing and automation engineering. It achieves detection and localization of moving targets by using edge detection, image matching, Kalman filtering, and other techniques, and it has the characteristics of being non-contact and fast, with high accuracy, and low testing costs [6]. The DIP technique generally requires setting a fixed target and tracking the position of the target to measure the structural deformation. The DIC method, on the other hand, is a full-field, lossless, non-contact photomechanical method that can adapt to both targeted and untargeted test conditions to achieve non-contact real-time deformation testing at the 0.01 sub-pixel level [7,8,9]. Although the DIP-based sub-pixel deformation testing method has lower testing accuracy than the DIC, its algorithm is simple, fast, low-cost, and easy to embed in smart hardware for edge computing, so it is suitable for most structural health-monitoring application scenarios.

DIP-based non-contact measurements involve edge-detection algorithms, which focus on extracting edge pixels with large grayscale variations in the target image. The edge detection operator identifies the pixel-level edges of an image using first- or second-order derivative operators, and thus the edges are judged by the grayscale variations within the neighborhood of the image pixels [10,11,12]. The existing popular edge detection operators, such as the Roberts operator [13], Laplacian operator [14], Prewitt operator [15], Sobel operator [16], and Canny operator [17], can achieve edge detection of images at the pixel level. Among them, the Canny operator uses non-maximal suppression and morphological connectivity operations [17], so that it detects the complete edges and has better edge continuity, which is widely used in image edge detection [18]. Moreover, in terms of sub-pixel level edge-detection algorithms, Lyvers [19] proposed spatial moments to detect image edges. This method computes the four parameters: the edge is rotated clockwise by *φ*. Meanwhile, the parameters *k* and *l* represent the precision of edge detection and the coarseness of edges of the model using six spatial moments, which is computationally intensive. However, spatial moments are polynomial functions and do not have orthogonality, thus generating redundant information in image processing. Ghosal et al. [20] proposed the edge detection method using Zernike orthogonal moments, i.e., they calculated the four parameters of the model by the Zernike moments of three different orders of the image, and then determined the image edges based on them. Zhao [21] derived 9 × 9 Zernike moment template coefficients based on the original 5 × 5 Zernike moments, which improved the accuracy but greatly increased the computational effort.

Prior research has indicated that performing displacement calculations with a low camera acquisition frame rate or excessively large displacement, resulting in significant inter-frame displacement, may impede the efficacy of the digital image correlation (DIC) algorithm. In such cases, the algorithm may require prolonged calculation times or fail to achieve accurate results. To expand the range of displacement recognition within traditional DIC algorithms, the prevalent approach is to broaden the search area of the entire pixel search algorithm. However, this course of action entails a rapid escalation in computational complexity and memory footprint. Addressing these challenges, this study proposed a new displacement measurement method based on the digital image processing (DIP) algorithm and validates it through experimentation.

**Figure 1:**
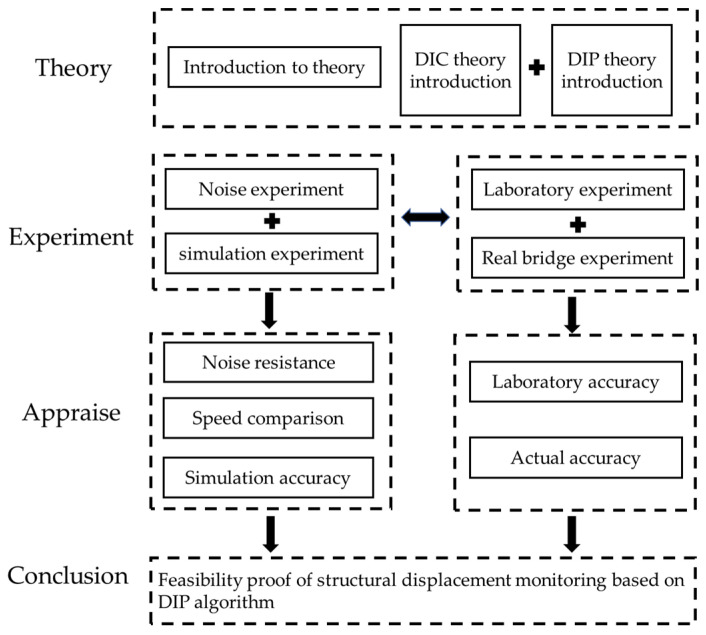
The structure of this article.

In this paper, a comparative study on the accuracy of structural displacement testing based on edge detection and DIC is conducted to verify the feasibility of edge detection-based algorithms in practical engineering applications. The structure of this article is shown in Figure 1. First, this paper briefly introduces the displacement test method based on the Canny operator and Zernike moment. Second, the target pattern with sub-pixel displacement is generated by computer simulation, and the displacement is calculated by applying DIC, Canny, and Zernike moment, respectively. Finally, the computational accuracy and computational efficiency of the algorithms are compared. Under laboratory conditions, this paper obtained accurate displacement results using a stepper displacement test system, and also applied the above three methods to process the stepper displacement images and compares them with the test results. In the end, this paper compared the structural displacement test results in a real bridge scenario with those of the conventional linear-displacement transducer.

## 2. Testing Principle of Edge Detection and DIC

### 2.1. Displacement Testing Principle Based on Edge Detection

**Figure 2:**
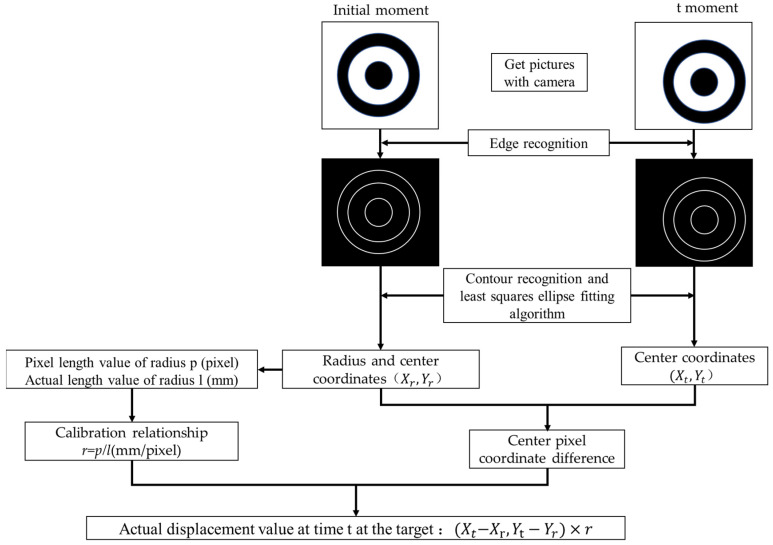
Schematic Diagram of Edge Detection Test Principle.

The detailed idea of the edge-detection algorithm for displacement testing is shown in Figure 2. First, the initial moment image is obtained by the camera, and then the outline of the target is acquired via the edge-detection algorithm. Further, the concentric circle center of the target is located by edge detection and least-squares ellipse-fitting algorithm to obtain the pixel length of the concentric circle radius. Next, the simple self-calibration of the camera is implemented in combination with the known geometry of the target. Finally, the center coordinates of the target at time t are obtained in the same way, and the difference of the center coordinates is calculated and combined with the calibration results to realize the displacement test based on the edge-detection algorithm.

At present, the classical detection operators of edge-detection algorithms at the whole-pixel level are the Roberts operator, Sobel operator, Canny operator, Laplace operator, etc. In this paper, we used the optimal detection operator, i.e., Canny operator [22], and the detailed process of the algorithm is as follows: (1) filter and smooth grayscale images using a two-dimensional Gaussian filter [17]; (2) compute the gradient magnitude and direction using the Sobel operator; (3) apply maximum suppression to the image gradient magnitude; and (4) detect and connect edges using a double-thresholding algorithm. After edge detection by the Canny operator, as shown in Figure 2, the displacement is calculated by an ellipse-fitting algorithm with three concentric circles, that is, conducting least-squares fitting on the basis of edge detection, whose algorithm accuracy can reach sub-pixel level [23]. 

**Figure 3:**
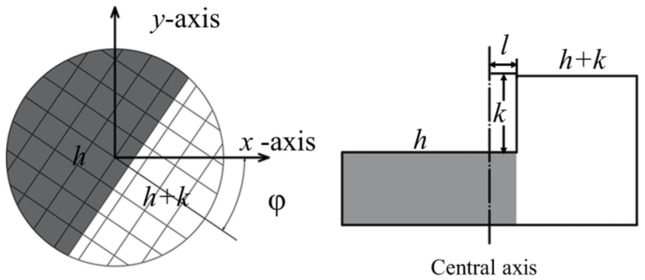
The step model of Zernike moment.

The main methods for sub-pixel level edge-extraction algorithms are the moment method, fitting method, and interpolation method. In order to prioritize the accuracy results, the sub-pixel edge-detection algorithm based on Zernike moments [24] was chosen in this paper. Its step model is shown in Figure 3, and there are four relevant parameters, where *k* is the grayscale difference of the step edge, characterizing the recognition accuracy; *h* represents the grayscale value of the background side; l denotes the minimum distance from the origin to the edge, which is used to characterize the thickness of the edge; and *φ* is the angle between the edge and the *x*-axis. The formula for calculating the four parameters of a pixel point in a grayscale image is as follows.

(1) $$A_{00}{}^{\prime }=A_{00}\;=h\pi +\frac{k}{2}\pi -k\sin^{-1}(l)-kl\sqrt{1-l^{2}}$$

(2) $$A_{11}{}^{\prime }=A_{11}e^{j\varphi }=\frac{2k}{3}{\left(1-l^{2}\right)}^{\frac{3}{2}}$$

(3) $$A_{20}{}^{\prime }=A_{20}=\frac{2k}{3}l{\left(1-l^{2}\right)}^{\frac{3}{2}}$$

(4) $$l=\frac{A_{20}}{A^{\prime }{}_{11}},\;k=\frac{3A_{11}}{2{\left(1-l^{2}\right)}^{\frac{3}{2}}}$$

where $A_{00}$ and $A_{11}$ denote the Zernike moments of each order, $A_{11}{}^{\prime }$ is the complex moment, and $A_{20}$ is the result of $A_{20}{}^{\prime }$ after the edge is rotated clockwise by *φ*. Meanwhile, the parameters *k* and *l* represent the precision of edge detection and the coarseness of edges, respectively.

The Zernike moment operator uses the image’s all-order orthogonal moments to obtain two of the four parameters of each pixel to determine whether it is a sub-pixel edge point. Its sub-pixel coordinates can be calculated by the following equation:

(5) $$\left[\begin{array}{l} x_{s}\\ y_{s} \end{array}\right]=\left[\begin{array}{l} x\\ y \end{array}\right]+\frac{Nl}{2}\;\left[\begin{array}{c} \cos(\varphi )\\ \sin(\varphi ) \end{array}\right]$$

where *N* is the template size.

In this paper, a 5 × 5 Zernike moment operator is chosen to obtain the sub-pixel edges of the three-concentric circle localization hole. The center coordinates of the circle in the target and the radius size are obtained by ellipse fitting. Then, the pixel displacement value of the target is obtained according to the change of the circle center coordinate value before and after deformation. Subsequently, the camera parameters are calibrated by combining the relationship between the simulated radius and the real radius of the circle, and the real displacement of the target is finally obtained.

### 2.2. The Digital Image Correlation (DIC) Technology

The DIC algorithm is generally divided into two phases in the target search process: whole-pixel search and sub-pixel search. Whole-pixel search algorithms include point-by-point search [25], hill-climbing search [26], cross search [27], Fast Normalized Cross-Correlation (FNCC) [28], Fourier transform-based cross-correlation (FTCC) [29], etc., while sub-pixel algorithms contain mainstream algorithms such as forward additive Newton–Raphson (FN-AR) [30], forward additive Gauss–Newton (FN-GN) [31], inverse compositional Gauss–Newton(IC-GN) [32]. In this paper, the combined algorithm of FNCC + IC-GN was used to calculate the sub-pixel displacement value in which the sub-pixel interpolation is conducted by dual cubic interpolation. 

**Figure 4:**
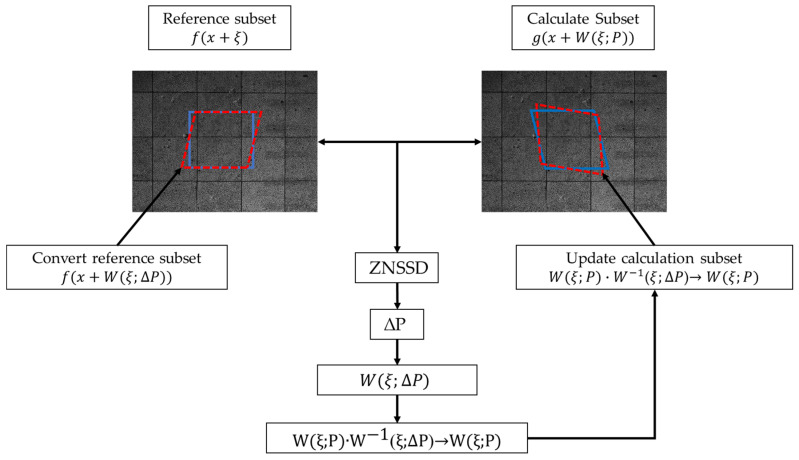
Schematic diagram of DIC test principle.

The DIC method [32] calculates the correlation based on the sub-regions in the two images before and after the deformation. As shown in Figure 4, a rectangle of size (2M+1)×(2M+1) is selected as the reference sub-region in the pre-deformed image centered on the computation point to be solved. In the deformed image, a rectangular region of the same size is traversed by some path, and then the correlation coefficient between the reference sub-region and the target sub-region is calculated by the Zero-mean normalized sum of squared difference (*ZNSSD*) correlation criterion [33] to obtain the whole-pixel displacement calculation result as:

(6) $$C_{ZNSSD}=\sum_{2M+1}\sum_{2M+1}{\left(\frac{f_{x}-\bar{f}}{\sqrt{\sum_{2M+1}\sum_{2M+1}{\left(f_{x}-\bar{f}\right)}^{2}}}-\frac{g_{x}-\bar{g}}{\sqrt{\sum_{2M+1}\sum_{2M+1}{\left(g_{x}-\bar{g}\right)}^{2}}}\right)}^{2}$$

where $f_{x},g_{x}$ are the grayscale values of the reference subset and the computed subset, respectively, and $\bar{f},\bar{g}$ are arithmetic means of $f_{x},g_{x}$, separately.

Afterward, we derive the *ZNSSD* equation to obtain the change in the deformation function as:

(7) $$\Delta p=-H^{-1}\times \sum_{\xi }\left\{{\left(\nabla f\frac{\partial W}{\partial p}\right)}_{6\times 1}^{T}\times [(f(x+\xi )-\bar{f}\right)-\frac{\Delta f}{\Delta g}(g(x+W(\xi ;p))-\bar{g}]\}$$

where H is the Hessian matrix, which can be calculated in advance, thus reducing the complexity of the algorithm, and improving the computational efficiency.

Finally, an iterative correction can be made to obtain the displacement results of the calculated points when the mode of Δp is less than a given threshold (typically 0.001). The calculation equation is presented as follows.

(8) $$W(\xi ,p)\leftarrow W(\xi ;p)\cdot W^{-1}(\xi ;\Delta p)=\left[\begin{array}{ccc} 1+u_{x} & u_{y} & u\\ v_{x} & 1+v_{y} & v\\ 0 & 0 & 1 \end{array}\right]{\left[\begin{array}{ccc} 1+\Delta u_{x} & \Delta u_{y} & \Delta u\\ \Delta v_{x} & 1+\Delta v_{y} & \Delta v\\ 0 & 0 & 1 \end{array}\right]}^{-1}$$

where $p=[u,u_{x},u_{y},v,v_{x},v_{y}]$ *u* and *v* are the displacements in the *x* and *y* directions, respectively, $\Delta p=[\Delta u,\Delta u_{x},\Delta u_{y},\Delta v,\Delta v_{x},\Delta v_{y}]$ and the iteration is considered to converge when |Δp| is less than 0.001.

This section provides a detailed theoretical introduction to the entire process of displacement calculation using the DIP and DIC algorithms. However, the practical performance of the algorithms needs further investigation, and performance evaluation indicators can provide a quantitative analysis of the algorithms’ performance. The next section will introduce the important evaluation indicators for the algorithm’s performance.

## 3. Error Evaluation

According to error analysis theory [34,35], the computational error of a sub-pixel displacement measurement algorithm can be represented by the mean error, standard deviation, and root mean square error. Assuming that the true value of the displacement (the reading of the micrometer) is $d_{read}$ and the displacement obtained by some algorithm [35] is $d_{mean}$, the mean error of the algorithm can be defined as

(9) $$E_{sys}=d_{mean}-d_{read}$$

where $d_{mean}$ is the arithmetic mean of the calculated displacement results at *N* points.

The standard deviation reflects the dispersion of the arithmetic mean of the calculated results and truly reflects the random error of the algorithm. It is denoted as

(10) $$\sigma =\sqrt{\frac{1}{N}\sum_{i=0}^{N}{\left(d_{mean}-d_{read}\right)}^{2}}$$

The root mean square error (*RMSE*) is the square root of the ratio of the square of the deviation of the predicted value from the true value to the number of observations *N*. It is used to measure the deviation between the predicted and true values and is expressed as

(11) $$RMSE=\sqrt{\frac{1}{N-1}\sum_{i=0}^{N}{\left(d_{\mathrm{mean}}-d_{read}\right)}^{2}}$$

This section introduced the evaluation indicators for the algorithm, mainly including the mean error that characterizes the algorithm’s accuracy and the *RMSE* that characterizes the algorithm’s stability. The factors that affect whether the algorithm is applicable are noise contamination, identification accuracy, and computation speed. The next section will comprehensively analyze and evaluate the performance of these algorithms based on experimental test, laboratory test and field test.

### 3.1. Translation Validation

**Figure 5:**
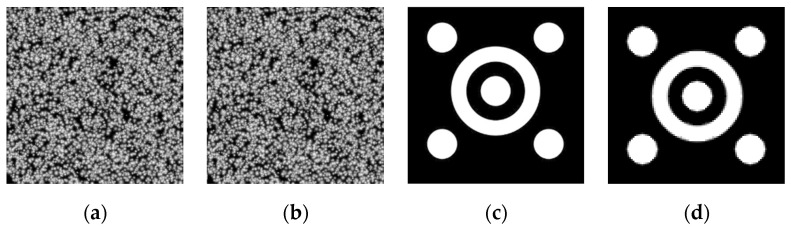
Target map for simulation experiment; (**a**) Original picture of DIC simulation experiment; (**b**) Translate down 1 pixel; (**c**) Original pictures of DIP simulation experiment; (**d**) Pan down 1 pixel.

**Figure 6:**
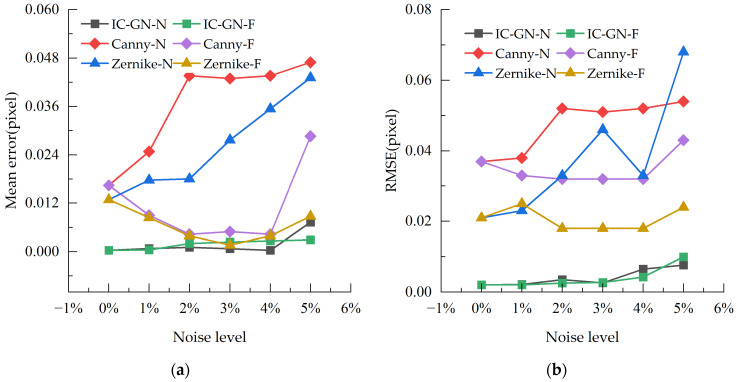
Performance evaluation of three algorithms (where N is before filtering and F is after filtering): (**a**) error variance; and (**b**) root mean square error.

To verify the feasibility of the above edge-detection and DIC testing methods, simulation experiments for error analysis were conducted in this paper. A computer algorithm was utilized to generate one scatter map suitable for DIC calculation (see Figure 5a,b). In the meantime, this scatter map was translated based on the fast Fourier transform (FFT), where the translation range is 0~1 pixel and the step size is 0.01 pixel, and finally, a set of 101 images in total was obtained. Subsequently, Gaussian white noise was applied to this group of images with noise levels of 1%, 2%, 3%, 4%, and 5% to obtain six groups of experimental images, respectively. Then, the IC-GN algorithm was calculated for these six groups of images, and Gaussian low-pass filtering was applied to the five noise groups [36], followed by IC-GN calculation for the filtered images to obtain the pre and post filtering results. To verify the noise sensitivity of the DIP algorithm, the images used for DIP calculation in this paper were adopted with three concentric circle targets [37] (see Figure 5c,d), while the FFT was employed to translate this image, where the translation range was 0~1 pixel and the step size is 0.01 pixel. It is worth noting that the simulated target pattern used by the author here (Figure 5c,d) is not a binary image. Finally, a set of 101 images in total was obtained. In the same manner, Gaussian white noise was applied to this set of images with noise levels of 1%, 2%, 3%, 4%, and 5% to obtain six sets of experimental images, but this time, the Canny algorithm and the edge-detection algorithm based on Zernike moments were computed separately for these six sets of images. After the Gaussian filtering, another calculation was performed to obtain the results before and after the filtering. Figure 6 illustrates the mean error and root mean square error of each algorithm before and after the filtering.

As can be seen from Figure 6, the mean error and *RMSE* of all three algorithms reach the highest at 5% noise level, so the data at 5% noise level are analyzed for the three algorithms. In the experimental results, the mean error of the DIC algorithm is 0.007 pixel and 0.002 pixel after filtering; the *RMSE* is 0.09 pixel and 0.099 pixel after filtering. Thus, we can infer that Gaussian filtering can improve the accuracy of the IC-GN algorithm, but not much for stability. In addition, the mean error of the Canny algorithm is 0.047 pixel and 0.029 pixel after filtering; its *RMSE* is 0.054 pixel at maximum and 0.043 pixel after filtering. Hence, it can be learned that Gaussian filtering has a more limited performance improvement for the Canny algorithm. Furthermore, the mean error of the Zernike moment algorithm is 0.043 pixel and 0.008 pixel after filtering; its *RMSE* is 0.043 pixel and 0.024 pixel after filtering. Therefore, it is clear that Gaussian filtering is more obvious for the Zernike moment algorithm.

In summary, the DIC algorithm based on IC-GN has the best noise immunity; while among the edge-detection algorithms, the Canny algorithm and the Zernike moment algorithm are more sensitive to the behavior of both added noise and filtering. Moreover, the performance of the Zernike moment algorithm is enhanced by Gaussian filtering, and relatively, the Canny algorithm is improved but with limited gains. Therefore, this demonstrates that selecting a Gaussian filter can effectively boost the algorithm’s performance.

### 3.2. Simulation Experiments

In order to avoid the influence of the systematic error of the camera, off-surface error, brightness variation, and other factors on the measurement, this paper conducts a comparative study on the accuracy of the DIC algorithm, Canny edge detection, and displacement recognition based on Zernike moments using three concentric circular targets (see Figure 5) in simulated experiments. Before deformation, the size of the baseline image is 400 × 400 pixels. The target image before deformation is translated 100 times along the Y-direction using the bicubic B-spline interpolation method, with a step size of 0.1 pixel for each translation. So, 100 groups of 100 target images after deformation are generated. Figure 5c presents the target images before deformation, and Figure 5d is the image after downward translation by 1 pixel.

**Figure 7:**
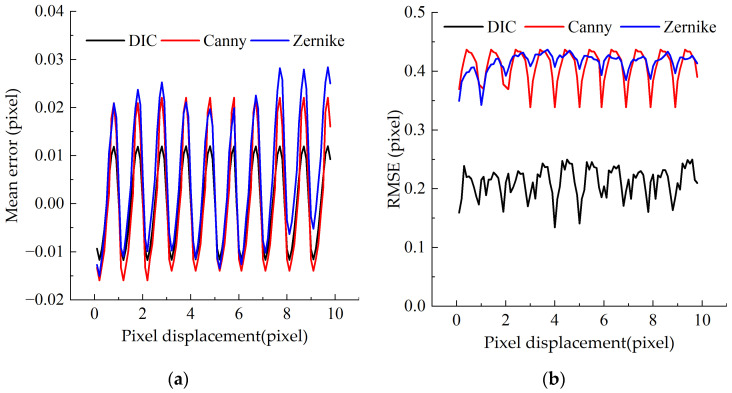
Performance evaluation of simulation experiment algorithm: (**a**) displacement—error mean diagram; and (**b**) displacement-error mean square diagram.

Figure 7a shows the mean error of the three algorithms, and it can be observed that the error of the DIC algorithm is the smallest, which is no more than 0.03 pixel. While the errors of the Canny and Zernike moment algorithms are greater, both reaching 0.06 pixel, and there is no significant difference. Besides, the error of the DIC algorithm is mostly spread between ±0.02 pixel, while the error values of the Canny and Zernike algorithms are between ±0.03 pixel. In Figure 7a, the periodically varying interpolation error that arises is due to the bicubic B-spline interpolation approach, which produces a sub-pixel level displacement of the simulated image, resulting in a periodic characteristic of the error as well. As can be obtained from Figure 7b, the *RMSE* of the DIC algorithm is about 0.2 pixel, while that of the Canny algorithm and the Zernike algorithm is about 0.4 pixel. It can be seen that the stability of the Canny algorithm and Zernike moment algorithm is similar, while the stability of DIC algorithm is better than all of them.

To sum up, it is obvious that the DIC algorithm has the best error accuracy and stability, and its accuracy error is 0.03 pixel. The Zernike moment is slightly better than the Canny algorithm. The accuracy error of them is 0.06 pixel. The pixel errors of all three algorithms are less than 0.1 pixel.

**Figure 8:**
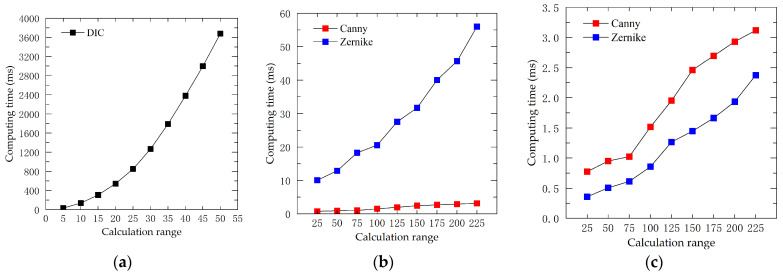
Algorithm speed comparison: (**a**) DIC algorithm; (**b**) DIP before CUDA acceleration; and (**c**) DIP after CUDA acceleration.

In order to evaluate the performance of the three displacement calculation methods more comprehensively, the computational speeds of different algorithms were compared based on the same computer hardware configuration. Then, a set of 100 computed images of 600 × 600 pixels was generated and applied to the three algorithms to compute sub-pixel displacements. Figure 8a shows the time consumption (in milliseconds) of a single point calculation with different search ranges (DIC integer pixel FNCC algorithm). Figure 8b shows a comparison of the time consumption (in milliseconds) of the two algorithms for a single point with different search ranges (edge-detection algorithms). Figure 8c shows the time consumption (in milliseconds) of two different search ranges (edge-detection algorithms) for a single point after CUDA acceleration. Taking the search range of 50 pixels between the DIC and DIP algorithms as an example, it can be seen from Figure 8a,b that the time spent to calculate a single point is 3678 ms; 0.95 ms for the Canny algorithm, and 12.91 ms for the Zernike moment algorithm. It can be noted that the Canny algorithm and Zernike moment algorithm are significantly faster than the DIC algorithm, and the complexity of Zernike moment algorithm is mainly due to the computation of multiple convolutions. Meanwhile, this study performed Compute Unified Device Architecture (CUDA) parallel acceleration for the Zernike moment algorithm with NVIDIA GTX950M as the graphics card. The results are displayed in Figure 8c. Before CUDA acceleration, the Canny algorithm are much faster than the Zernike moment algorithm in speed. However, with CUDA parallel acceleration, the computation speed of the Zernike moment algorithm is increased from 56.01 ms to 2.37 ms in in a search range of 225 pixels, nearly 24 times faster, making it comparable to the Canny algorithm.

### 3.3. Experimental Validation

**Figure 9:**
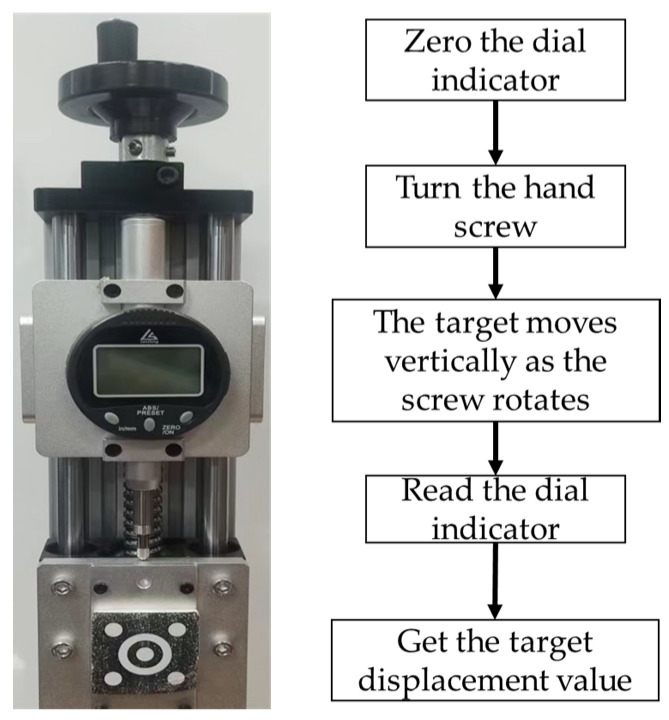
Step displacement measuring system.

In this section, we perform an experimental verification of the proposed algorithm. As shown in Figure 9, the experiment is based on a step displacement test system, which consists of a micrometer, a hand screw, a slider, etc. The target is a rectangle with a size of 4 × 4 cm, and three concentric circles are adopted for the pattern. It is worth mentioning that during the experiment, the target is attached to the slider. In the measuring process, the hand screw moves the slider with the rotation of the screw; when the slider moves, the micrometer is operated to measure the distance moved by the slider as the real displacement value. An 8-megapixel industrial camera (Fantasia Technology, FT-U3880, resolution: 3964 × 2228 pixel; lens focal length: 16 mm) is employed for video acquisition and video processing. Then, the generated results are compared with the micrometer readings for error analysis.

**Figure 10:**
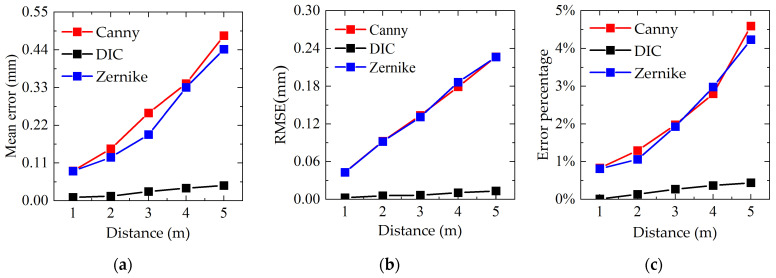
Performance evaluation of laboratory experimental algorithms: (**a**) mean error; (**b**) error variance; and (**c**) variation of error percentage with distance.

The experiment adopts five distance variables of 1 m, 2 m, 3 m, 4 m, and 5 m, and does not replace the lens. The loading is conducted at 2 mm as one level, with a total of 5 levels, and the micrometer readings during the movement are recorded. Therefore, the experimental calibration results are 0.147 mm/pixel, 0.297 mm/pixel, 0.451 mm/pixel, 0.602 mm/pixel, and 0.786 mm/pixel in order from 1 m to 5 m. Figure 10a plots the average error versus distance. As can be seen from the figure, with the increase in distance, the mean error of each algorithm increases, and the value and increase rate of the DIC algorithm are lower than those of the Canny and Zernike moment algorithms. Further, Figure 10b provides the variation of the mean squared error with distance. It is clear that the mean squared error of the DIC algorithm is smaller than that of both the Canny and Zernike algorithms, and with increasing distance, the mean squared error grows much less rapidly than the other two algorithms. Besides, Figure 10c shows the error percentage (the ratio of the maximum value of error to the maximum w-shift value) versus distance. It is obvious that the percentage error of all three algorithms increases as the distance increases. The DIC algorithm has the smallest increase in error percentage, while the Zernike and Canny algorithms do not differ significantly, with an error of less than 5%.

Under laboratory conditions, the mean error and the *RMSE* of the DIC algorithm at 1 m distance are 0.01 mm and 0.003 mm; those of the Canny algorithm are 0.089 mm and 0.043 mm; and those of the Zernike moment algorithm are 0.086 mm and 0.043 mm. DIC has a better algorithm mechanism, so its accuracy is higher than the other two algorithms. Canny algorithm is an integer pixel-level operator, which can achieve sub-pixel accuracy through fitting algorithm. Zernike moment algorithm is a sub-pixel operator, and its theoretical accuracy and actual accuracy are slightly higher than Canny algorithm. In summary, under laboratory conditions, there is not much difference between the Canny algorithm and the Zernike moment algorithm in terms of measurement accuracy and stability; the DIC algorithm is better than both of them.

### 3.4. Real Bridge Experiments

**Figure 11:**
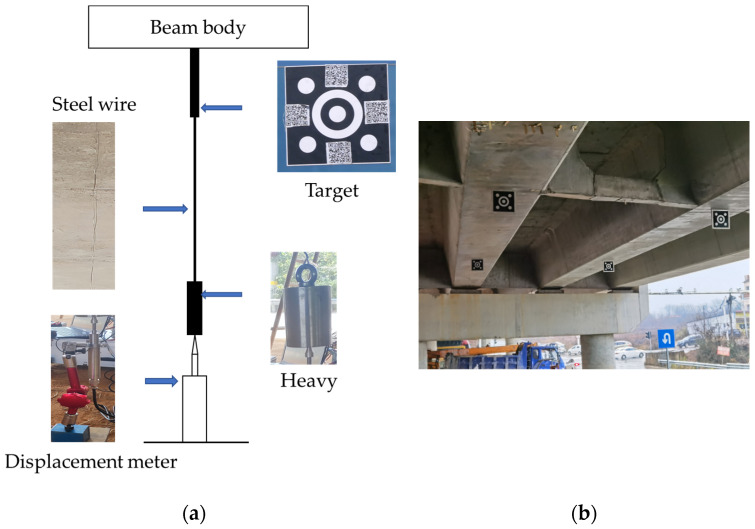
Site conditions of real bridge experiment: (**a**) site layout of the bridge test: (**b**) site target layout.

To verify the practicality of the above visual measurement method, we conducted real bridge experiments. The field test took a continuous small box girder vehicular bridge with 30 m span diameter of prestressed concrete as the test object in Yuelu District, Changsha City, China. As shown in Figure 11a, the test system is deployed at the bottom of the midspan of the bridge span during the video displacement test, which uses Linear Variable Displacement Transducer (LVDT, Donghua Test, model: DN5921; accuracy: 0.005 mm) for the bridge dynamic deflection test, and the error analysis of the video displacement detection system is performed considering its test results as the real value. In addition, as shown in Figure 11b, solid targets for deformation testing are set in the bridge span, and concentric circle markers for edge-detection algorithms are pasted on the targets. To ensure the accuracy of the DIC algorithm, some 2D codes are pasted on the side of the three concentric circle targets as the feature texture available for DIC–algorithm testing. It is notable that the civilian SLR (single-lens reflex) camera (Panasonic Lumix GH5, resolution 3840 × 2160, 100~400 mm zoom lens) for the video test is set up on the horizontal ground about 20 m away from the target area, and the focus and angle of the camera are adjusted. A lead hammer weight is suspended by a wire below the target steel plate, and an LVDT sensor is connected below the weight, whose acquisition frequency is 50 Hz and test time is 600 s. All instruments and parameters for the real bridge experiment are shown in Table 1. The experiment can be divided into three steps. First, the video of the target area during the vehicle passing over the bridge is recorded by a SLR camera, while the dynamic displacement of the bridge is collected by LVDT, and finally compared with the video displacement measurement results. Three video segments of about 15 s each (exactly the time for a heavy vehicle to pass over the entire bridge span) are intercepted from the recorded video. Therefore, the calibration results of the three working conditions are working condition 1: 0.3829 mm/pixel, working condition 2: 0.3549 mm/pixel, and working condition 3: 0.3052 mm/pixel.

**Figure 12:**
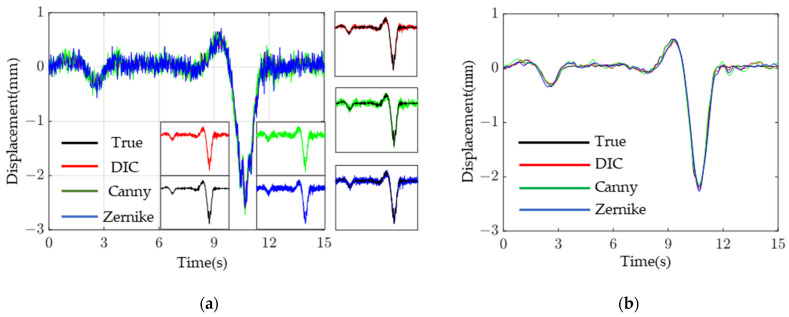
Displacement time history curve: (**a**) before EMD filtering; and (**b**) after EMD filtering.

**Figure 13:**
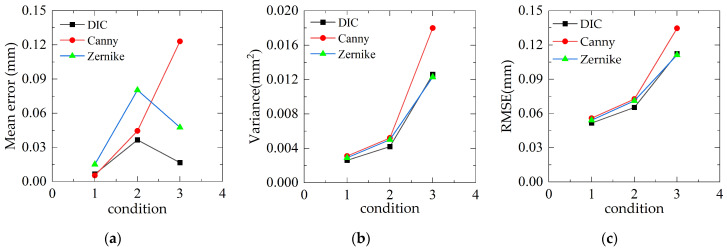
Performance evaluation of real bridge experiment algorithm: (**a**) mean error; (**b**) variance; and (**c**) mean square error.

Figure 12a shows the test results comparison of the working conditions and bridge-span displacement time course for a heavy vehicle crossing the bridge. For convenient for comparing, the empirical mode decomposition (EMD) is deployed to perform high-frequency filtering on the displacement time-range data, and the results are shown in Figure 12b. As can be seen from the figure, the three vision-based testing algorithms agree well with the results of LVDT. The peak deflection and error of the bridge obtained by the four displacement measurement methods under three working conditions are shown in Table 2. Additionally, Figure 13 compares the results of the maximum error, error variance, and root mean square error of the displacement time course curves of the three displacement measurement methods relative to the LVDT displacement test under each test condition. As can be seen from Figure 13 and Table 2, in the real bridge experiment, the maximum error value of DIC algorithm is 0.036 mm and its maximum *RMSE* is 0.111 mm in three working conditions; the maximum error value of Canny algorithm is 0.123 mm and its maximum *RMSE* is 0.134 mm; the maximum error value of Zernike moment algorithm is 0.081 mm and its maximum *RMSE* is 0.112 mm. It can be seen that the DIC algorithm test results are better than Canny and Zernike moment algorithms in terms of accuracy and stability; The accuracy and stability of the Zernike moment algorithm is slightly better than that of the Canny algorithm. Therefore, although the error and stability of the two algorithms based on DIP are not as good as those of the DIC algorithm, they are also within a controllable range and are acceptable in engineering.

## 4. Conclusions

The non-contact deformation-measurement system based on digital image correlation (DIC) algorithm has been widely utilized in structural deformation-testing in civil engineering. However, it presents certain limitations in specific scenarios, such as large frame-to-frame displacement. These limitations are characterized by two specific issues: (1) sub-pixel accuracy of the DIC algorithm cannot converge normally when the actual sampling frequency is low, which requires the recalculation of the entire pixel to provide an initial value; and (2) in the case of significant deformation of the tested structure, an enlarged search range is required to achieve accurate tracking and identification, resulting in a significant increase in computational time.

To address the limitations of non-contact deformation measurement systems in the aforementioned special scenarios, the digital image pattern (DIP) was introduced. Through comparative analyses of the DIC and DIP algorithms in terms of testing accuracy, noise resistance, and operational speed via a simulation experiment, laboratory test, and field test, the effectiveness of the DIP algorithm in civil engineering deformation testing was validated. The study provides a basis for the application of non-contact testing systems in special scenarios.

The main conclusions of this study are drawn as follows:

(1) The whole-pixel operator Canny and sub-pixel operator Zernike moments were introduced to investigate the testing accuracy and stability of the DIP displacement measurement. The results showed that the two operators had little difference in accuracy and stability;
(2) In the presence of large frame-to-frame displacement, to ensure the robustness of the DIC algorithm, it is necessary to perform a whole-pixel search for initial values. In such cases, the Canny and Zernike moment algorithms have significant advantages in terms of computation speed and cost compared to the DIC algorithm.
(3) The computation speed of the Canny algorithm was higher than that of the Zernike moment algorithm. When CUDA-based parallel acceleration was applied to the latter, the computation speeds of these two algorithms were comparable;
(4) Field test results showed that the testing errors of the DIC method, Canny algorithm, and Zernike moment algorithm were all less than 0.15 mm. However, the DIC algorithm exhibited a more obvious advantage in testing accuracy;
(5) This study introduced the DIP theory and proposed an algorithm for structural displacement testing, which can compensate the limitation of the increased computational cost of the DIC algorithm in the presence of large frame-to-frame displacement testing.

## Figures and Tables

**Table 1:** List of hardware equipment used in the real bridge test.

Equipment	Parameter	Value
Displacement sensor (DN5921)	accuracy	0.005 mm
	Sampling frequency	50 Hz
Camera (Lumix GH5)	resolution	3840 × 2160
	Framerate	50 Hz
Camera lens	focal length	100–400 mm zoom

**Table 2:** Maximum value and error of bridge displacement.

Test Method	Maximum Displacement			Maximum Displacement Time Error		
	Condition I	Condition II	Condition III	Condition I	Condition II	Condition III
Displacement meter	−1.7813	−2.1852	−1.8669			
DIC	−1.7748	−2.2217	−1.8504	0.0065	−0.0365	0.0165
Canny	−1.776	−2.1408	−1.99	0.0053	0.0444	−0.1231
Zernike	−1.7663	−2.2656	−1.9144	0.015	−0.0804	−0.0475

## Data Availability

Not applicable.
